# A Repeated State of Acidification Enhances the Anticariogenic Biofilm Activity of Glass Ionomer Cement Containing Fluoro-Zinc-Silicate Fillers

**DOI:** 10.3390/antibiotics10080977

**Published:** 2021-08-13

**Authors:** Traithawit Naksagoon, Shoji Takenaka, Ryoko Nagata, Maki Sotozono, Tatsuya Ohsumi, Takako Ida, Naoki Edanami, Takeyasu Maeda, Yuichiro Noiri

**Affiliations:** 1 Division of Cariology, Operative Dentistry and Endodontics, Faculty of Dentistry & Graduate School of Medical and Dental Sciences, Niigata University, Niigata 951-8514, Japan; naksagoon-ttw@dent.niigata-u.ac.jp (T.N.); lemmings@dent.niigata-u.ac.jp (R.N.); sotozono@dent.niigata-u.ac.jp (M.S.); tatsuya.ohsumi@matsumidai-dc.com (T.O.); tida@dent.niigata-u.ac.jp (T.I.); edanami@dent.niigata-u.ac.jp (N.E.); noiri@dent.niigata-u.ac.jp (Y.N.); 2 Research Centre for Advanced Oral Science, Faculty of Dentistry & Graduate School of Medical and Dental Sciences, Niigata University, Niigata 951-8514, Japan; maedat@dent.niigata-u.ac.jp

**Keywords:** oral biofilm, glass ionomer cement, antibiofilm effect, dental material, fluoride ion, zinc ion

## Abstract

This study aimed to evaluate the anticariogenic biofilm activity of a novel zinc-containing glass ionomer cement, Caredyne Restore (CR), using a flow-cell system that reproduces Stephan responses. *Streptococcus mutans* biofilms were cultured on either CR or hydroxyapatite (HA) discs mounted on a modified Robbins device. The media were allowed to flow at a speed of 2 mL/min for 24 h while exposed to an acidic buffer twice for 30 min to mimic dietary uptake. Acid exposure enhanced biofilm inhibition in the CR group, which showed 2.6 log CFU/mm^2^ in viable cells and a 2 log copies/mL reduction in total cells compared to the untreated group after 24 h of incubation, suggesting enhanced anticariogenic activity due to the release of fluoride and zinc ions. However, there was no difference in the number of viable and total cells between the two experimental groups after 24 h of incubation in the absence of an acidic environment. The anticariogenic biofilm activity of CR occurs in acidic oral environments, for example in the transient pH drop following dietary uptake. CR restorations are recommended in patients at high risk of caries due to hyposalivation, difficulty brushing, and frequent sugar intake.

## 1. Introduction

Oral biofilms are communities of bacteria embedded within an extracellular matrix, creating a highly organized structure on hard dental tissue [1,2,3]. The presence of mature biofilms can lead to the development of dental caries. Frequent consumption of dietary sucrose provides a substrate for extracellular polysaccharide production and organic acid synthesis by acidogenic microorganisms [4]. A constantly acidic environment reduces microbial diversity and increases the acidic microbiota, causing dysbiosis. If the biofilm persists on the tooth surface, the interface between the tooth and the biofilm causes demineralization, prompting the development of a carious lesion [1,4]. To prevent this process, there are three main approaches, focusing on virulence (biofilm), host, and lifestyle factors. In terms of the virulence factor, mechanical elimination, such as professional tooth cleaning, tooth brushing, and flossing, are fundamental for controlling oral biofilms, and surgical intervention is unnecessary for removal [5]. Chemical controls, such as antimicrobial agents in toothpastes and mouthwashes, are used as alternative or adjunctive methods [5]. Dental restorative materials containing antimicrobials are also effective in preventing bacterial adhesion [2]. Strategies that promote host defenses can enhance resistance against decalcification by bacteria. Fluoride, for instance, can slow the progression of caries lesions on tooth surfaces, enhancing host defense via hydroxyfluoroapatite formation [6,7]. A strategy that has recently attracted attention involves maintaining the resilience of the oral microbiota by controlling the frequency of sugar intake [8].

The use of glass ionomer cement (GIC) remains one of the best choices for minimally invasive dentistry and house call dentistry due to its favorable fluoride release and uptake characteristics, chemical bonding to the tooth structure, and lower susceptibility to moisture [9,10]. GIC controls both caries progression adjacent to fillings and caries incidence. Fluoride interferes with cariogenic bacteria, disturbing bacterial colonization and affecting bacterial metabolism by inhibiting the glycolytic enzyme enolase and proton-extruding ATPase [10,11]. Thus, fluoride interacts with both the host (tooth) and the resident oral microflora. However, many studies have found that the amount of fluoride released from GIC is not effective enough to prevent biofilm formation, inhibit acid production by oral biofilms [10,12], or reduce the progression of caries along the tooth-restoration interface [13]. Therefore, additional antimicrobial compounds and nanoparticles have been added to the GIC to enhance its cariostatic effect [14].

Caredyne Restore (CR; GC Corporation, Tokyo, Japan), a recently launched GIC, contains a fluoro-zinc-silicate glass (BioUnion^TM^, GC Corporation, Bunkyo-ku, Tokyo, Japan) filler in addition to the fluoro-alumino-silicate glass [15,16,17]. Zinc ions inhibit the acid production activity of *Streptococcus mutans* by interfering with bacterial glycolysis and growth [18,19]. It has also been reported that the combination of zinc and fluoride ions disrupts the synthesis of insoluble glucans by *S. mutans*, inhibiting biofilm formation [20]. In fact, compared to conventional GIC, CR remarkably inhibited biofilm formation by *S. mutans* by interfering with bacterial adhesion [15]. One striking aspect was the high release of fluoride and zinc ions from the fluoro-zinc-silicate glass portion of the CR under acidic conditions [16,17]. In our previous report, the amount of fluoride ions released from the CR at pH 4.5 was twice that at pH 7.0 [16]. Surprisingly, the quantity of zinc ions released at pH 4.5 was 33-fold greater than that at pH 7.0 [16]. Thus, accurate values of ions released from the CR may be reflected during transient pH decreases, for example at approximately 30 min after dietary uptake. However, there are no studies that estimate the anticariogenic biofilm efficacy of CR while simulating pH fluctuations.

The purpose of this study was to evaluate the anticariogenic biofilm activity of CR under acidic conditions using a flow-cell system. Our study model, which used a modified Robbins device (MRD) flow cell system equipped with acid buffer exchange, enabled us to investigate whether acidic pH enhances anticariogenic biofilm properties.

## 2. Results

### 2.1. Scanning Electron Microscopy (SEM) Observations

**Figure 1:**
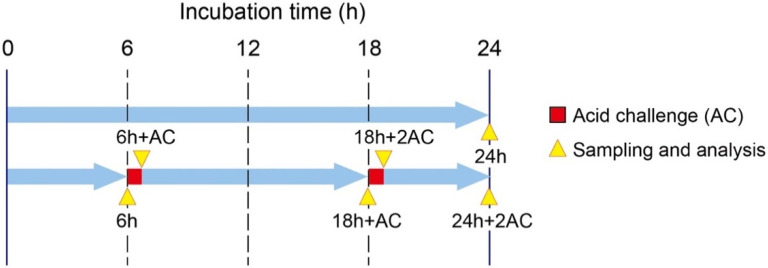
Experimental design showing the time schedule, treatment, and sampling. Acid challenge (AC) refers to exposure to 1M acetic acid (pH 4.5) for 30 min. Incubation and acid challenge were performed at a flow rate of 2 mL/min. Sampling and analysis were performed at the points indicated by yellow arrow heads. 6 h: 6 h of culture; 6 h + AC: A single exposure of acetic acid for 30 min after 6 h of incubation; 18 h + AC: The specimen was exposed to acidic solution after 6 h of incubation and then cultured up to 18 h; 18 h + 2AC: A second exposure of acetic acid after 18 h of incubation; 24 h: 24 h of culture without acidic treatment; 24 h + 2AC: The specimen was exposed to acidic solution after 6 h and 18 h of incubation during 24 h of culture.

**Figure 2:**
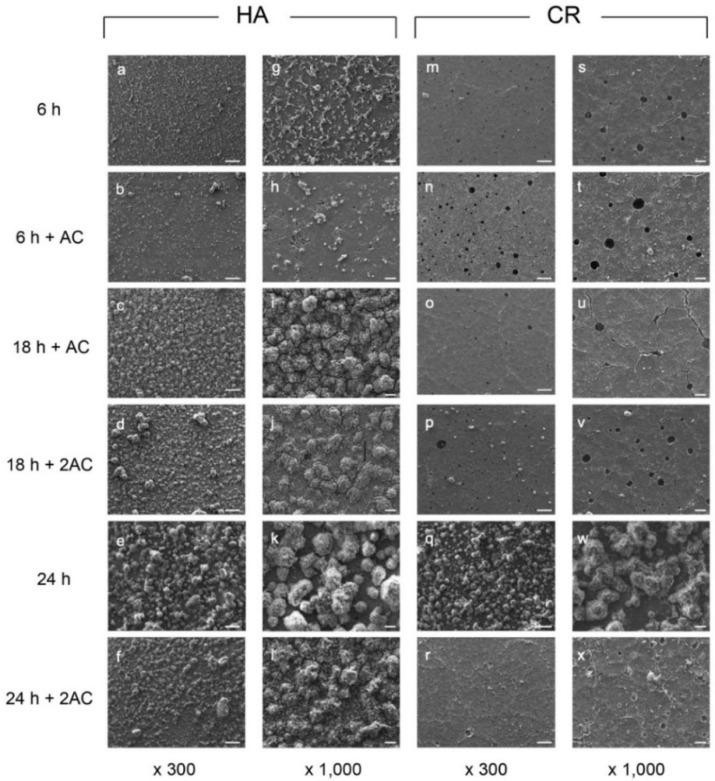
Representative SEM images of *S. mutans* biofilm formed on the HA (**a**–**l**) and the CR (**m**–**x**) at defined incubation times. 6 h: 6 h of culture; 6 h + AC: A single exposure of 1M acetic acid (pH 4.5) for 30 min after 6 h of incubation; 18 h + AC: The specimen was exposed to acidic solution after 6 h of incubation and then cultured up to 18 h; 18 h + 2AC: A second exposure of acetic acid after 18 h of incubation; 24 h: 24 h of culture without acidic treatment; 24 h + 2AC: The specimen was exposed to acidic solution after 6 h and 18 h of incubation during 24 h of culture; AC: acid challenge; AC2: acid challenge twice. Scale bars = 100 μm (×300), 20 μm (×1000).

The biofilm formation on each specimen with defined incubation times and treatments (Figure 1) is shown in Figure 2. The number of biofilm clusters in the CR group (Figure 2m,s) was less than that in the hydroxyapatite disc (HA; Olympus, Tokyo, Japan) group after 6 h of culture (Figure 2a,g). However, when incubated for 24 h without acidic treatment, specimens from both groups showed a similar number of biofilms (Figure 2e,k,q,w).

Single exposure to acidic solution at 6 h of incubation did not degrade the biofilm structure formed on the HA (Figure 2a,b,g,h). The number of biofilms on the HA increased during the culture for another 12 h following exposure to the acidic solution (Figure 2c,i). The second exposure to acidic solution at 18 h of incubation did not degrade the biofilm structure on the HA (Figure 2d,j). The number of biofilms on the HA after culturing for 24 h was approximately the same, regardless of the presence or absence of the acidic solution (Figure 2e,f,k,l). These findings indicated that acidic treatment on the HA did not affect biofilm development.

The biofilms formed on the CR while exposed to the acidic solution were sparse throughout the field of view (Figure 2n–v). The number of biofilms formed on the CR for 24 h (Figure 2r,x) when exposed to two acidic environments was much lower than that in the unexposed environment (Figure 2q,w). These findings indicated that CR has enhanced antibiofilm activity under acidic conditions.

### 2.2. Confocal Laser Scanning Microscopy (CLSM) Observation

**Figure 3:**
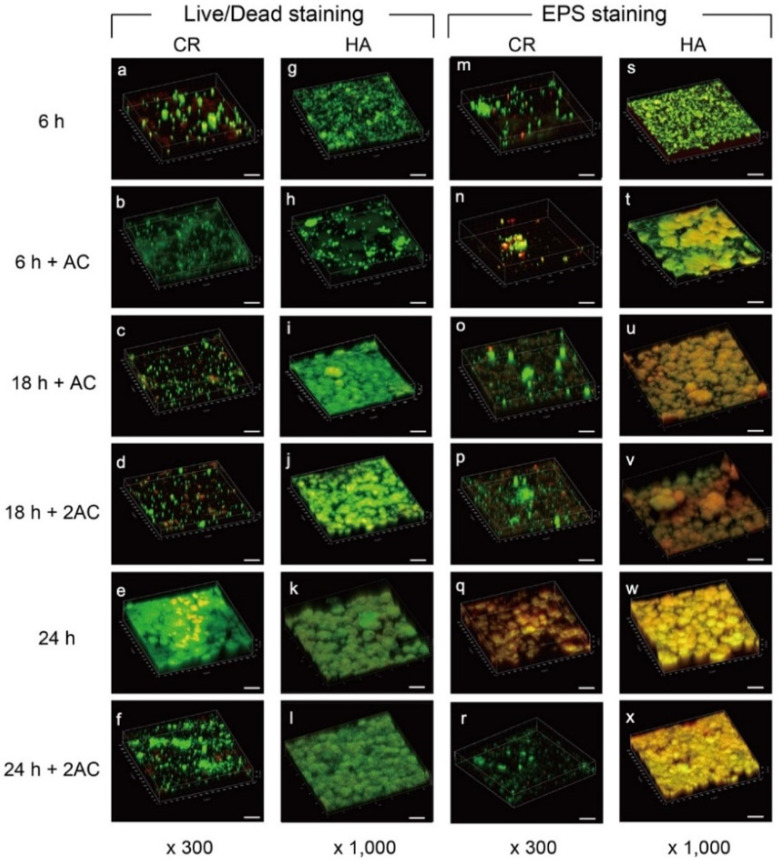
Representative 3-dimensional reconstructed images of *S. mutans* biofilm using Live/Dead (**a**–**l**) and SYTO9/rhodamine-B (**m**–**x**) staining. Live cells are stained green (SYTO9), and dead cells are stained red (propidium iodide). EPS staining was performed using SYTO9 for all bacteria and rhodamine-B for EPS (red). 6 h: 6 h of culture; 6 h + AC: A single exposure to 1M acetic acid (pH 4.5) for 30 min after 6 h of incubation; 18 h + AC: The specimen was exposed to the acidic solution after 6 h of incubation and then cultured up to 18 h; 18 h + 2AC: A second exposure to acetic acid after 18 h of incubation; 24 h: 24 h of culture without acidic treatment; 24 h + 2AC: The specimen was exposed to acidic solution after 6 h and 18 h of incubation during 24 h of culture. CR: Caredyne Restore; HA: hydroxyapatite; EPS: extracellular polymeric substances; AC: acid challenge; AC2: acid challenge twice. Scale bars = 50 μm.

Three-dimensional reconstructed images by CLSM at the defined incubation times and treatments (Figure 1) are shown in Figure 3. The number of bacteria (Live/Dead staining) and the quantities of extracellular polymeric substances (EPS staining) in the CR group (Figure 3a,m) were less than those in the HA group after 6 h of culture (Figure 3g,s). However, when incubated for 24 h without acidic treatment, the bacterial counts and biofilm volumes on the specimens from both groups appeared to be similar (Figure 3e,k,q,w). Some (but not all) bacteria that adhered to the CR surface after a 6 h incubation were dead, (Figure 3a), indicating that the CR cannot disinfect bacteria on the surface completely, with subsequent biofilm formation (Figure 3e,q). The microorganisms in the biofilm formed on the HA were mostly viable with or without exposure to an acidic environment (Figure 3g–j), indicating that *S. mutans* was resistant to acidity.

A single exposure of acidic solution after 6 h of incubation suppressed bacterial growth and EPS production on the CR after the subsequent 12 h of culture (Figure 3b,c,n,o), whereas biofilm formation on the HA was greatly promoted (Figure 3h,i,t,u). Similarly, a second exposure of acidic solution after 18 h of incubation inhibited biofilm formation on the CR after the subsequent 6 h of culture (Figure 3d,f,p,r). The bacterial count and the quantities of EPS formed on the CR for 24 h when exposed to the two acidic environments (Figure 3f,r) were much lower as compared to the unexposed environment (Figure 3e,q). These findings indicate that fluoride and zinc ions released from the CR by the acidic treatment inhibited bacterial growth and EPS production.

### 2.3. Viable and Total Cell Counts

**Figure 4:**
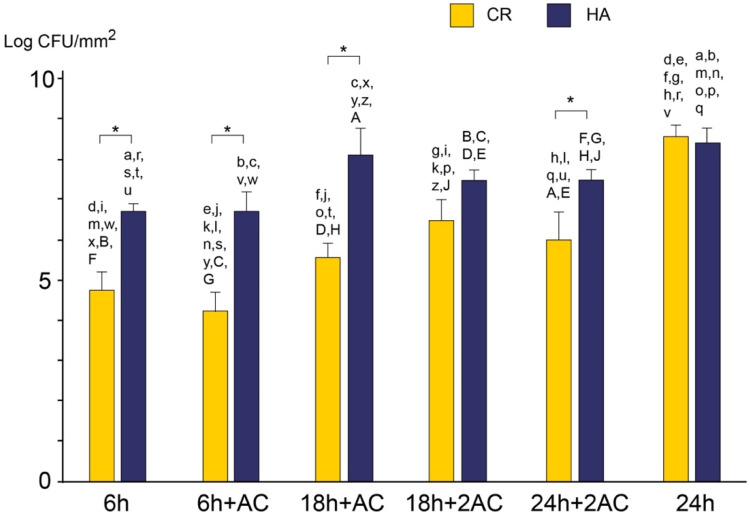
Viable counts of *S. mutans* biofilm cells after incubation at each defined time as determined by colony counts. The results are shown as means ± the SD of 5 replicates. Pairs of the same scripts represent significant differences (*p* < 0.05). * *p* < 0.05. 6 h: 6 h of culture; 6 h + AC: A single exposure to 1M acetic acid (pH 4.5) for 30 min after 6 h of incubation; 18 h + AC: The specimen was exposed to the acidic solution after 6 h of incubation and then cultured up to 18 h; 18 h + 2AC: Second exposure of acetic acid after 18 h of incubation; 24 h: 24 h of culture without acidic treatment; 24 h + 2AC: The specimen was exposed to acidic solution both after 6 h and 18 h of incubation during 24 h of culture. CR: Caredyne Restore; HA: hydroxyapatite; AC: acid challenge; AC2: acid challenge twice.

The number of viable cells with defined incubation times and treatments is shown in Figure 4. There were significant differences between the CR and HA groups in four sampling periods: 6 h, 6 h + AC, 18 h + AC, and 24 h + 2AC (*p* < 0.05). Although CR effectively prevented bacterial adhesion at 6 h, bacteria developed on both CR and HA to the same extent after 24 h without any acid exposure. Acid exposure inhibited the biofilm formation in the CR group, revealing a 2.6 log CFU/mm^2^ reduction compared to the untreated group after 24 h incubation, whereas there was no significant difference between 24 h + 2AC and 24 h in the HA group. The numbers of bacteria after incubation for 24 h, including the two acid challenges in the CR group, were not significantly different when compared with those of 6 h, 18 h + AC, and 18 h + 2AC. An acid challenge did not induce any remarkable change in cell viability, nor did it cause detachment of the biofilm developed on either experimental specimen.

**Figure 5:**
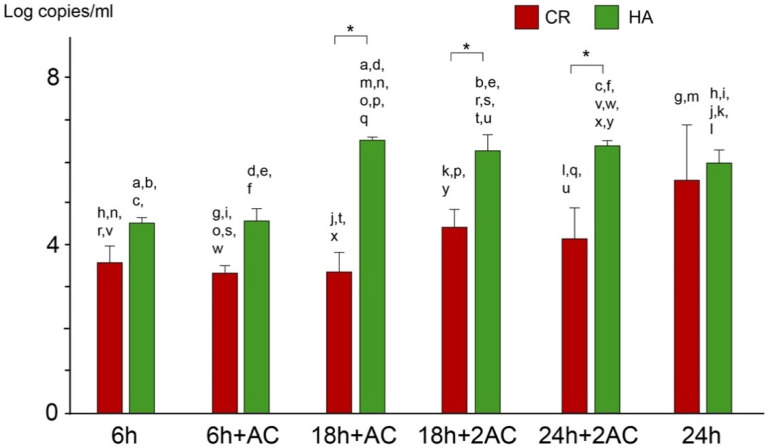
Total bacterial counts of *S. mutans* biofilm cells after incubation at each defined time as determined by colony counts. The results are shown as means ± the SD of 5 replicates. Pairs of the same scripts represent significant differences (*p* < 0.05). * *p* < 0.05. 6 h: 6 h of culture; 6 h + AC: A single exposure to 1M acetic acid (pH 4.5) for 30 min after 6 h of incubation; 18 h + AC: The specimen was exposed to the acidic solution after 6 h of incubation and then cultured up to 18 h; 18 h + 2AC: A. second exposure of acetic acid after 18 h of incubation; 24 h: 24 h of culture without acidic treatment; 24 h + 2AC: The specimen was exposed to acidic solution after 6 h and 18 h of incubation during 24 h of culture. CR: Caredyne Restore; HA: hydroxyapatite; AC: acid challenge; AC2: acid challenge twice.

The total cell count showed a similar tendency as the viable cell count (Figure 5). There were significant differences between the CR and HA groups at three sampling periods, including 18 h + AC, 18 h + 2AC, and 24 h + 2AC (*p* < 0.05). The cells in the biofilm that grew while the CR was treated with two acids decreased by 2 log copies/mL compared to that of the group without any acid exposure.

There was no significant difference between the CR and HA groups after 24 h of incubation without any acid challenge. The numbers of bacteria remaining in the CR group after incubation for 24 h, including the two acid challenges, were similar to the numbers of bacteria remaining after 6 and 18 h, respectively (*p* > 0.05). An acid challenge did not cause detachment of the biofilm developed on either experimental specimen.

## 3. Discussion

Dental caries represent a biofilm-mediated, dynamic disease. They are multifactorial in nature, and excess sugars are a primary cause [21]. Acidogenic bacteria in dental biofilms metabolize dietary fermentable carbohydrates and produce organic acids. The decrease in pH in the oral environment is known as the Stephan curve [22]. The pH value rapidly decreases to a maximum of 4.5 within 10 min, and it takes 30 to 60 min to recover to its starting value [23]. Since this pH fluctuation occurs daily, an in vitro experimental model for estimating the anticariogenic biofilm activity of dental materials needs to consider pH fluctuations.

In this study, the anticariogenic activity of CR was evaluated after exposure to an acidic solution once or twice for 30 min during biofilm formation, mimicking the pH drop following dietary uptake. *S. mutans* developed on both the CR and HA after 24 h in the absence of an acid exposure, showing no significant differences in viable cell and total cell counts (Figure 4 and Figure 5). The number of viable cells that adhered to the CR was significantly less than that on the HA after culturing for 6 h without any acid exposure, indicating that the anticariogenic activity of CR only occurs immediately after mounting under neutral pH conditions. The MRD flow cell system supplied fresh medium continuously to flush out organic acid produced by bacteria and maintain the pH in the chamber at 7.0 [16].

**Figure 6:**
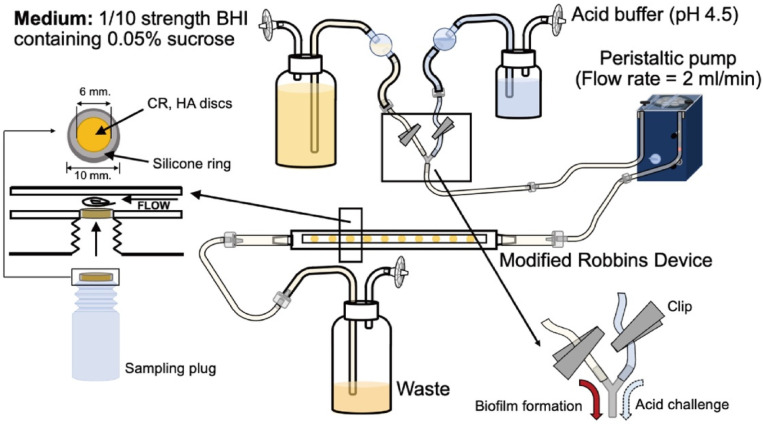
Modified Robbin device used in this study.

The acidic treatment enhanced the anticariogenic biofilm activity in the CR group. The total bacterial count after 6 h of culture (Figure 5; 6 h and 6 h + AC) showed no significant difference compared to 18 h + AC, 18 h + 2AC, and 24 h + 2AC, respectively. Furthermore, there was no significant difference in viable counts between the 6 h and 24 h + 2AC groups. In contrast, the total bacterial count after 24 h of culture, including the two acid challenges (Figure 5; 24 h + 2AC), was significantly higher than after 6 h of culture (Figure 5; 6 h and 6 h + AC). In addition, viable and total bacterial counts on the HA grown while being exposed to the acidic solution twice (Figure 5; 24 h + 2AC) were equivalent to those in the 24 h culture without any acid challenge. These findings suggest that enhanced ion release from CR in an acidic environment effectively inhibits biofilm formation.

The enhanced antibiofilm activity of CR in acidic environments is useful not only in transient decreases in pH during dietary uptake, but also in patients at high risk of caries due to radiation exposure, hyposalivation, xerostomia, difficulty brushing, and frequent sugar intake. In addition, root caries lesions, especially in interproximal spaces, are difficult to clean, so dental biofilms can develop easily without disturbance. Repeated states of acidification underneath the biofilm may be beneficial for CR.

It has been reported that CR increases the release of zinc and fluoride ions under acidic conditions [17]. Kohno et al. evaluated the ion release, recharge ability, and anticariogenic biofilm properties of CR using an in vitro saliva drop setting device [24]. The results showed that, with repeated exposure to acid over 7 days, the concentration of zinc ions remained at a level that consistently inhibited *S. mutans* and saliva-derived multi-species biofilm formation. It has also been shown that CR can recharge and release its ions on applying a tooth gel containing zinc and fluoride ions.

The flow cell system is a powerful tool for the in vitro evaluation of bacterial biofilms under fluid shear force while simulating the oral environment [25,26]. The system allows for the growth of mature biofilms, excluding the overgrowth of planktonic cells and accumulation of bacterial metabolites [25,26]. Although some types of flow cell systems have been used to investigate the anticariogenic biofilm activity of restorative materials [27,28,29], to the best of our knowledge, no model has reproduced the dynamic oral environment. In this model, an acidic solution was applied to the MRD device twice for 30 min by switching the medium supply. This model enabled us to analyze the anticariogenic biofilm effect of GIC when the oral cavity became acidic. However, this model has some limitations that need to be addressed.

Caries and periodontal disease are lifestyle-related and biofilm infections. Diet, salivary dysfunction, and poor oral hygiene can impact the equilibrium antagonistic and cooperative interactions in biofilms containing resident normal microflora, resulting in dysbiosis [4]. The pH inside the biofilm decreases gradually, and there may be multiple pH values within the biofilm. Rapid changes in acidic environments may not accurately reproduce the oral environment. In addition, the rapid drop in pH causes the death of non-acid-resistant bacteria. Furthermore, acid production is one of the many biological processes that occur within biofilms [23].

Another method for estimating the antibiofilm properties of dental restorative materials is an in vivo experiment using an oral appliance-mounted enamel or dentine specimen. This method includes a cariogenic challenge, which is an exposure phase to a sucrose solution several times during bacterial acidogenesis [30,31,32,33]. Padovani et al. evaluated the influence of different restorative materials, such as resin composite, GIC, and amalgam, on the biofilm structure by CLSM. The specimens were adapted to a palatal device, and the participants wore the device for 7 days. They were exposed to a cariogenic challenge. The biofilm parameters showed no statistical difference with respect to the different materials. However, GICs visually showed a prevalence of non-viable cells forming small clusters distributed by the biofilm [30]. Although the in vivo model closely reproduces the oral environment, care must be taken in interpreting the data due to individual differences, such as constituent bacterial species and saliva volume.

## 4. Materials and Methods

### 4.1. Specimen Preparation

CR and HA specimens were prepared as described previously (Table 1) [15,16]. Briefly, CR was mixed manually according to the manufacturer’s instructions, and the mixtures were packed in acrylic molds with a diameter of 6 mm and a thickness of 1.5 mm. The mold was pressed against 2 smooth glass slabs and stored for 1 h at 37 °C with a relative humidity of 100%. CR and HA of the same size and dimension were polished using 4000 grit silicon carbide paper (Marumoto Struers KK, Tokyo, Japan) under water. Then, all the samples were disinfected with 70% ethyl alcohol and washed twice with phosphate-buffered saline (pH 7.0). In total, 96 discs were prepared for each material group.

Specimens were attached on the sampling plugs of the MRD using a silicone ring (10 mm). The MRD was sterilized using ethylene oxide gas for 4 h [16]. The flow cell system consisted of a medium-and acid-buffered medium reservoir, peristaltic pump, and carboy for waste, all connected through silicone tubes (Figure 6).

### 4.2. Bacteria

*S. mutans* UA159 was used in this study, and the inoculum was prepared according to a previously published protocol [15]. A sterile saliva solution was prepared as described previously [15,16,34]. Briefly, unstimulated saliva was obtained from one of the authors. Saliva sample was diluted (1:10) with sterile Ringer solution containing 0.05% cysteine (Sigma-Aldrich, St. Louis, MO, USA). The diluted solution was then centrifuged for 10 min and the supernatant was filter-sterilized (pH = 6.8). Then, 20 mL of adjusted human saliva was pumped into an MRD chamber at a flow rate of 2 mL/min and kept static for 2 h at 37 °C to form the salivary pellicle layer on the surface of each specimen.

The bacterial suspension (optical density = 0.020–0.025 at 590 nm) was then pumped into the device and kept static for 30 min at 37 °C to acquire initial bacterial adhesion under anaerobic conditions.

### 4.3. Biofilm Formation and Acid Challenge

The medium, which was 1/10th the strength of brain heart infusion broth (BHI; Difco Laboratories, Detroit, MI, USA), was supplemented with 0.05% sucrose (pH = 7.0) and pumped into the device at a flow rate of 2 mL/min. Biofilms were allowed to develop anaerobically for a maximum of 24 h at 37 °C under continuous flow conditions. During biofilm formation, some experimental groups were exposed to an acidic solution at 6 and 18 h after the start of culturing. Either 1M acetic acid (pH 4.5) or PBS was pumped into the device for 30 min at a flow rate of 2 mL/min (Figure 1).

Samples were collected from the MRD at each defined time point. The acid buffer was flushed out with PBS for 10 min prior to sample collection. The collected specimens were washed twice with phosphate-buffered saline (PBS). They were then randomly divided into 4 groups for analysis, including morphological observations using either SEM or CLSM, viable cell counting, and total cell counting.

### 4.4. SEM Observation

Samples were prepared as previously described [15,16]. An SEM (EPMA-1610, Shimazu, Kyoto, Japan) was used to observe the biofilm structure at magnifications of ×300 and ×1000. Two discs were prepared for each experimental group. Three randomly selected fields per experimental group were observed at both magnifications.

### 4.5. CLSM Analysis

Bacterial viability in the biofilms and structures was observed using CLSM (LSM700, Carl Zeiss, Jena, Germany). Two samples per experimental group and condition were stained with Live/Dead BacLight bacterial viability kit (Live/Dead staining; Thermo Fisher Scientific, Waltham, MA, USA) according to the manufacturer’s instructions. Another 2 samples per experimental group and condition were subjected to EPS staining using SYTO9 and rhodamine-B (Wako Pure Chemical Industries Ltd., Osaka, Japan). Staining was performed as previously described [15,16]. When a combination of SYTO9 and rhodamine-B is used, SYTO9 stains all bacteria without their viability, and rhodamine-B reveals the extent of the biomass, or EPS. The filter settings were 510–530 nm for SYTO9 and more than 610 nm for propidium iodide and rhodamine-B using Ar 488 nm and He-Ne 543 nm lasers. Three-dimensional reconstructed images were created using the Imaris software (Bitplane AG, Zurich, Switzerland). The assay was performed with a total of three replicates for each experimental group and condition.

### 4.6. Viable Cell Counting

Viable cell counting was performed as described previously, with slight modifications [15,16]. Briefly, the biofilm was ultrasonicated for 5 min, shaken vigorously for 1 min, and ultrasonicated again for 5 min to detach the biofilm from the material surface. The bacterial suspension was homogenized, serially diluted, and plated on BHI agar plates. Colony-forming unit (CFU) counting was performed after anaerobic incubation for 48 h at 37 °C. This assay was performed with 5 replicates per treatment.

### 4.7. Total Cell Counting

Quantitative analysis of the total bacteria on the specimen was performed using the polymerase chain reaction-invader method (BML, Inc., Tokyo, Japan), as described previously [16]. This assay was performed with 5 replicates per treatment.

### 4.8. Statistical Analyses

Statistical analyses were performed using the Statistical Package for the Social Sciences (version 11.0 SPSS Inc., Chicago, IL, USA) and Excel Statistics 7.0 (Esumi Co., Ltd., Tokyo, Japan). When applicable, the data were presented as means ± standard deviation, and significant differences were determined using the Kruskal-Wallis test with a post hoc Steel-Dwass test. *p* < 0.05 was considered statistically significant.

## 5. Conclusions

Within the limitations of this in vitro study, the anticariogenic biofilm activity of CR occurs only in its early phases under neutral pH conditions. The release of fluoride and zinc ions from CR is enhanced in acidic oral environments, for example in the transient pH drop following dietary uptake. CR restorations are recommended, especially in patients who are at higher risk of caries due to radiation exposure, hyposalivation, xerostomia, the presence of difficult brushing sites such as interproximal root surfaces, and frequent sugar intake.

## Figures and Tables

**Table 1:** Materials used in this study.

Materials	Code	Lot No.	Composition
Caredyne Restore	CR	1809061	Powder: fluoro-alumino-silicate glass, fluoro-zinc-silicate glass, pigment Liquid: polyacrylic acid, distilled water, polybasic carboxylic acid
Hydroxyapatite	HA	170915	Hydroxyapatite

## Data Availability

Data sets used during the study are available from the corresponding author on reasonable request.
